# Computational Identification of Novel Amino-Acid Interactions in HIV Gag via Correlated Evolution

**DOI:** 10.1371/journal.pone.0042468

**Published:** 2012-08-03

**Authors:** Olga V. Kalinina, Heike Oberwinkler, Bärbel Glass, Hans-Georg Kräusslich, Robert B. Russell, John A. G. Briggs

**Affiliations:** 1 CellNetworks, Bioquant, University of Heidelberg, Heidelberg, Germany; 2 Department of Infectious Diseases, Virology, Universitätsklinikum Heidelberg, Heidelberg, Germany; 3 Structural and Computational Biology Unit, European Molecular Biology Laboratory, Heidelberg, Germany; University of South Florida College of Medicine, United States of America

## Abstract

Pairs of amino acid positions that evolve in a correlated manner are proposed to play important roles in protein structure or function. Methods to detect them might fare better with families for which sequences of thousands of closely related homologs are available than families with only a few distant relatives. We applied co-evolution analysis to thousands of sequences of HIV Gag, finding that the most significantly co-evolving positions are proximal in the quaternary structures of the viral capsid. A reduction in infectivity caused by mutating one member of a significant pair could be rescued by a compensatory mutation of the other.

## Introduction

Certain positions in protein or nucleic acid sequences show correlations in their mutation patterns during evolution. This phenomenon has been termed ‘correlated mutations’ or ‘co-evolution’. Co-evolution is typically defined by the statistical analysis of multiple sequence alignments.

Correlated mutations have been successfully exploited for prediction of RNA secondary structure, owing to the precise rules governing base-pairing of nucleic acids (e.g. [1]). Similar predictions can be made for extracellular proteins using disulphide bonds: cysteines in two positions that are observed to change simultaneously within particular sequences can be inferred to be bonded together.

The notion that other such pairings within protein sequences might exist has prompted much research over the past 20 years. The first attempts date back to 1994 [2], when correlated amino acid changes were proposed to assist 3D structure prediction, followed by many other approaches (e.g., [3]–[7]). However, the accuracy of predicting structural contacts in this way never exceeded 20% [8], even though correlated positions were found to be associated with diseases [7]. The overall difficulty in interpretation of the correlated positions is probably due to a lack of clear pairing rules for amino acids within protein structures, and because proteins maintain structural integrity over long evolutionary distances (e.g., [9]). Nevertheless, studies coupled to various experimental techniques show that co-evolving residues contribute to key aspects of protein function (e.g., [11], [12]).

A common problem of co-evolution based methods is a tendency to overpredict the significance of a correlation, due to the inability to account for the influence of random drift in ancestral lineages that fixed in the evolution of larger clades. For example, predicted co-evolving positions in the HIV *env* gene [6] correlate with functionally important protein properties, but the analysis identifies too many correlated sites (involving 263 of 764 positions in the protein) to be amenable for detailed structural analyses or experimental testing. Elaborate methods have been developed to cope with this problem (e.g., [10]) and have successfully been applied to such complicated systems as the HIV Env V3 loop. Recently, several studies report to have solved the problem of overprediction by building an underlying statistical model that explains all the observed correlations in the most economical way [13], [14]. However, most of these approaches are still rather computationally expensive.

In this study, we explore whether the larger datasets that are available with the new sequencing approaches can improve the sensitivity of the correlation-based methods. We apply co-evolution analysis to HIV Gag, a viral protein with a large number of closely related sequences that makes it one of the largest sets of rapidly evolving sequences available. Of the previously proposed approaches for the analysis of co-evolving positions, few are currently available and none of these can immediately handle new datasets involving many thousands of sequences. We implemented an approach similar to the mutual information method proposed recently by Gloor et al. [3] with suitable adaptations to handle large sequence datasets. We sought to see if the co-evolution approach could reveal new insights about HIV Gag structure and function, and to confirm the functional relevance of identified correlations by measuring virus fitness experimentally.

HIV Gag is a polyprotein, which forms the major structural component of the virus (reviewed in refs. [15], [17]). It consists of four major folded protein domains: the membrane binding MA domain, two domains which together make up CA, and the Zn finger-containing RNA-binding NC domain. These domains are connected by flexible linkers. HIV assembly proceeds through oligomerisation of Gag on the underside of the plasma membrane. Assembly is driven by MA-membrane-MA, CA-CA and NC-RNA-NC interactions. The assembling Gag lattice buds out through the plasma membrane to form the immature virus. During, or shortly after, budding, Gag is cleaved in five places by the viral protease leading to rearrangement of the virus particle into its mature, infectious form. Within the mature virus, CA assembles to form a characteristic cone-shaped capsid, NC is condensed within the RNP at the centre of the virus, MA remains associated with the viral membrane. The flexible nature of the inter-domain linkers in Gag has prohibited high-resolution structural studies on the complete molecule. However, X-ray and NMR derived structures are currently available only for individual protein domains. There is also a high-resolution structure available for CA oligomerised in its mature lattice form [17], but not in the immature lattice form. Beyond its role in assembly, Gag and its constituent domains have functions in recruiting cellular proteins for trafficking and budding, and in early events of the virus life cycle (reviewed in refs. [15], [18]). Gag is therefore a protein capable of carrying multiple functions, adopting multiple different arrangements, and forming multiple interactions with itself and with other cellular and viral proteins. Co-evolving residues might suggest the presence of inter or intramolecular Gag-Gag interactions, the movement of short linear motifs or other signaling sites, or mechanisms for conserving other functional surfaces.

## Results

An alignment consisting of 7396 Gag protein sequences, ranging from 39.5% to 99.8% identity with an average of 80.9%, was analyzed to identify pairs of amino acid positions showing a significant correlation, i.e. a high mutual information of the amino acid distributions, along with a low joint entropy to avoid positions that were conserved across all sequences. Significant pairs are usually those in which a sizeable population of sequences contains two particular amino acids at two positions and another population contains two other amino acids. A set of 65 pairs, spanning 36 positions was identified as significant. In each pair of positions, there are two major variants of the distribution of amino acids among the sequences, all the other variants being minor. For example, for the most significant hit, the pair 203E-301Y (here and elsewhere position numbering follows the sequence of GAG_HV1B1), the pattern E-Y appears in 48% of all sequences, and the pattern D-F in 49%, whereas the intermediate patterns, E-F and D-Y appear in only 0.6% and 2% of sequences respectively. We considered the two groups of sequences containing the E-Y and the D-F pairs. The analysis of the average distance from each sequence to other sequences of the same group and to both groups combined shows almost identical means and variances. Thus the substitution pattern cannot be explained by evolution from the common ancestor. Although one of the two variants is observed with a greater frequency in certain subtypes, they apparently have appeared more than once in the course of evolution.

**Figure 1:**
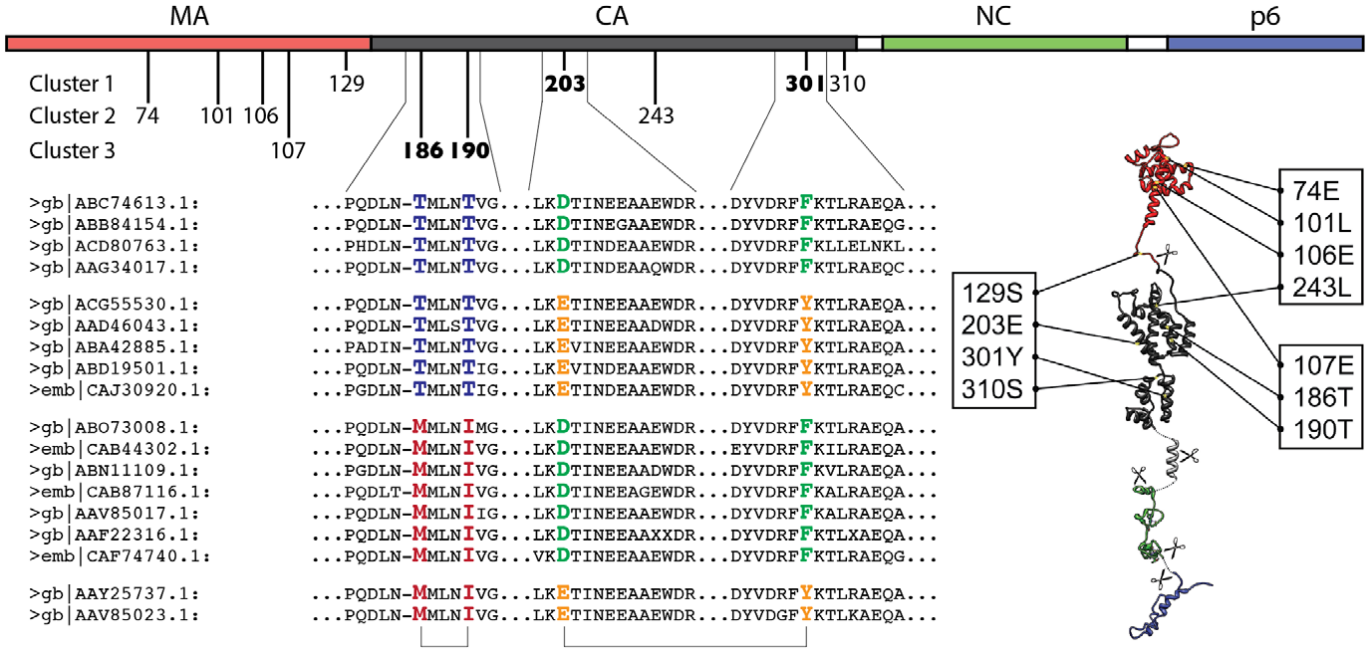
The location of the positions described in the text on Gag sequence and structure. The top shows a linear representation of Gag domain structure, with positions numbered according to GAG_HV1B1. The three non-trivial co-evolving clusters are shown beneath the domain structure, as are the appropriate regions of the alignment for a sub-set of the sequences used. Sequences are labeled by their GenBank (gb) or EMBL (emb) accession codes. The structures on the bottom right show where the positions in the figure lie on the known three-dimensional structures of Gag domains. The two most significant pairs are marked in the alignment.

The positions from all significant pairs fall into 8 clusters of mutually correlated positions. Two of these clusters separate HIV-1 from HIV-2 and SIV (as defined by taxonomic annotation in the NCBI nr database) and thus the correlation might be due to random drift after separation of these lineages. From the remaining six clusters, three (135V-139Q-141Q, 196A-198M, 358H-363L) are comprised of amino acids separated by fewer than four others in the sequence, so the correlation is likely the result of pressure on the amino acids neighboring in the structure. However, three of the identified clusters –74E-101L-106E-243L, 107E-186T-190T, and 129S-203E-301Y-310S – cannot obviously be explained by sequence proximity or divergence. These three clusters are illustrated in Figure 1. They include the two most significant pairs: 203E-301Y (49% D-F, 48% E-Y) and 186T-190T (71% T-T, 20% M-I).

The other pairs in these clusters show a much weaker degree of correlation (typically, the second most frequent amino acid pair appears in ∼1% of the sequences). An exception from this is the 129S-301Y pair (32% S-Y, 31% Q-F). At present, we cannot explain this interaction with any structural reasoning, but it is possible that these sites come in contact at some point during assembly. Interestingly, the 129S site is located close to the protease cleavage site between MA and CA, and thus might experience some specific evolutionary pressure.

We have performed similar analysis on 10 randomly chosen subsets, each consisting of 1/10^th^ of the sequences, of the whole dataset used in this study. When choosing the 65 top-scoring co-evolving pairs (the number of significant pairs found for the full dataset), the resulting sets include between 27 to 35 positions, which is similar in number to the 36 positions found from the whole set. However, the composition of the sets is different: between 17 and 23 of the individual positions overlap with the 36 found in the whole dataset, and between 21 and 36 of the co-evolving pairs overlap with the 65 found in the whole dataset. The overlap of the results between the subsets is similarly low. This suggests instability of the result for smaller subsets.

Nevertheless, the most significant 203E-301Y and 186T-190T pairs are present in the result for all subsets. The less significant 129S-301Y pair appears in only one subset. Other pairs from the three clusters do not appear in the result for the smaller subsets. We therefore elected to focus on the two most significant pairs.

The second most significant pair, 186T-190T, represents two amino acid residues separated by a single turn of an α-helix. As for the adjacent positions mentioned above, here the explanation is relatively simple, since the proximity of their side chain atoms (4.2 Å) makes interactions virtually inevitable.

**Figure 2:**
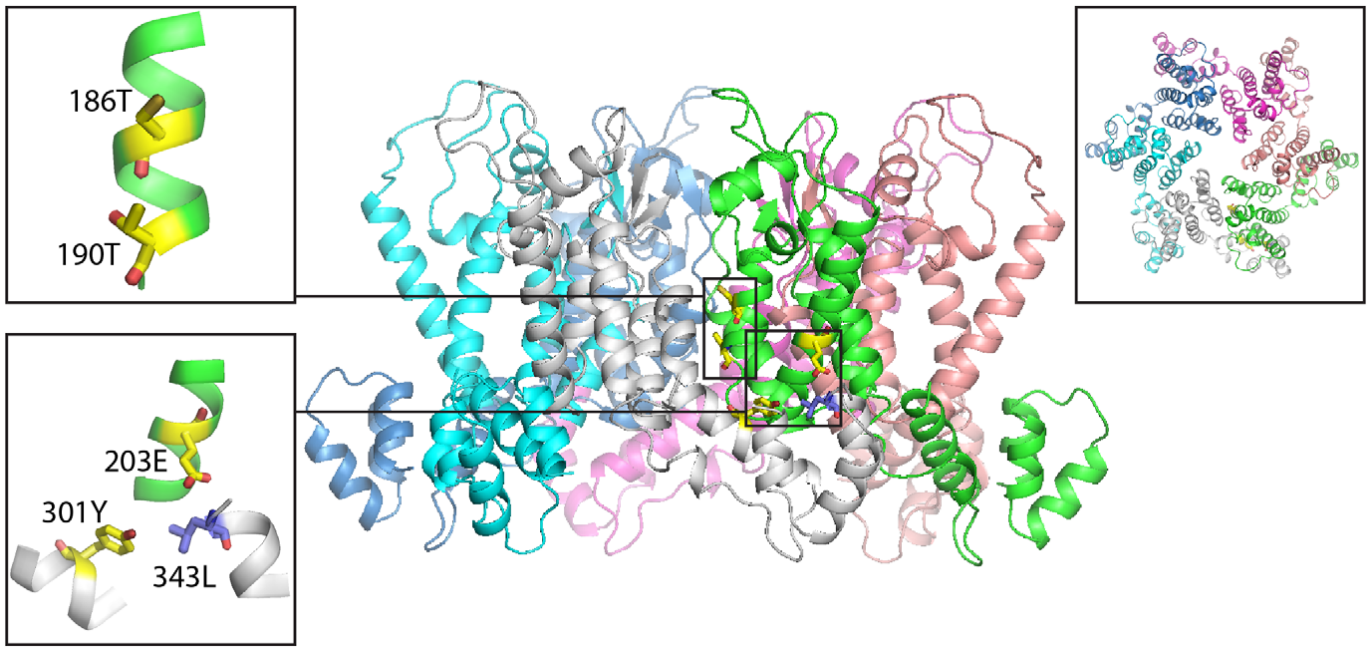
Spatial arrangement of the two top-scoring pairs in a side view of the hexameric unit-cell of the mature capsid lattice (PDB ID 3mge). A top view is shown in the top-right inset. The left insets highlight the top-scoring pairs of residues, which are shown in the sticks representation (yellow carbons). The side chain of 343L, which has previously been demonstrated to be crucial for formation of the mature lattice, is also shown (blue carbons).

The most significant pair –203E and 301Y – in contrast, contains two positions that are located far from one another both in sequence and in structure: 203E is located in the N-terminal domain of CA, whereas 301Y is located in the C-terminal domain of CA. They might nevertheless play an important role in the formation of the quaternary structure of the capsid. A recent X-ray structure of the hexagonal unit of the capsid [17] highlights an interface between the N-terminal domain and the C-terminal domain of the adjacent CA monomer in the hexameric ring. Within this structure, the side-chains of 203E in one monomer and 301Y in the adjacent monomer are separated by only 6.1 Å (Figure 2). The side chain of 343L projects into the gap between the two residues. It has previously been described that mutation of 343L to either S or A prevents formation of the mature CA lattice [19]. Mutation of 301Y to A also prevents mature lattice formation [19]. These strongly co-evolving residues therefore contribute to a critical interface between adjacent CA molecules within the mature CA lattice. This pair was also identified as a member of a network of phylogenetic associations, that includes 716 pairs [20].

Recently, it has been reported that building a statistical model that would explain all the observed correlation in the most parsimonious way [13], [14] could significantly improve the specificity, to an extent when reconstruction of a 3D structure of the protein becomes feasible. We have applied two such published methods, PSICOV [14] and EvFold [13] to our dataset. PSICOV was unable to handle a dataset of this size (memory errors). From the top-scoring pairs produced by EvFold that have a score greater or equal to 0.1 (27 in total) and are more than two amino acid positions apart, we identify the most significant 203E-301Y and 186T-190T pairs, as well as a number of other pairs that were not identified by our approach. A closer inspection shows that they are either very conserved, or the amino acid changes only in one site and stays the same in the second, thereby not conforming to our assumption that the intermediate variants are unviable. Thus we observe a good agreement of our results and those obtained from another method.

**Figure 3:**
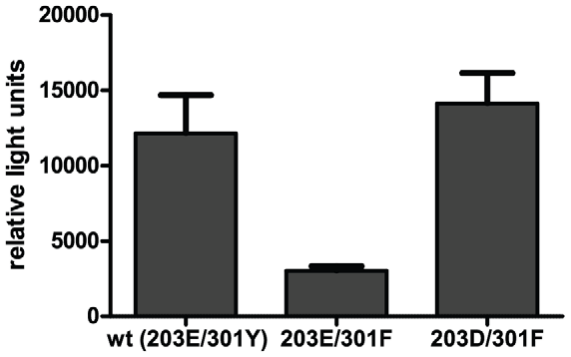
The effect of changes in the most significant co-evolving pair on virus infectivity. The reduction in infectivity caused by the single mutation is rescued by the compensatory mutation in the co-evolving residue.

We set out to test experimentally the effect of single and double mutations in this pair of positions on viral fitness. We hypothesized that if two residues strongly co-evolve, then a minor variant should be less fit than the major variants. To test this hypothesis we constructed full-length HIV-1 plasmids carrying either the wild type sequence 203E-301Y, a change in one of the co-evolving residues or in both. Viral particles obtained by transfection of the respective proviral plasmids were subsequently analysed for virus infectivity (Figure 3). The single change 203E-301F led to a fourfold drop in virus infectivity compared to wild-type. Introducing a further change in the other co-evolving residue to give the second major variant 203D-301F restored infectivity close to wild-type levels, supporting our hypothesis. This result suggests that the evolutionary pressure leading to co-evolution of these two residues results directly from an effect on viral fitness when only one residue is mutated.

## Discussion

Co-evolving positions have long been proposed to have functional importance in the protein structure. Approaches to identify co-evolving positions have been extensively tested previously [12], demonstrating that the approaches can often identify positions close in space, but normally with poor sensitivity and specificity. Here we show that using a large dataset of diverged sequences for the specific case of fast evolving viral sequences can identify new co-evolving mutations that have structural and functional significance.

Extensive studies over the years have provided tens of thousands of viral sequences representing a unique source of data for statistical studies. Viral proteins experience a strong positive evolutionary pressure that leads to substantial divergence over short time while largely conserving function and structure. Community efforts such as the 1000 genomes [21] and Genome 10 K [22] projects will also provide thousands of sequences from other species. These sequences will probably have properties different to those of virus sequences, partially due to the complicated biology of the organisms considered and partially due to the different nature of the evolutionary pressure that acts upon them. Nevertheless, when studied with methods like that described here, these data will open up new exiting possibilities for the use of previously developed techniques to identify potentially interesting positions for structure and function.

## Materials and Methods

### Dataset and Alignment

The sequences were extracted by comparing the Gag sequence from Uniprot accession GAG_HV1B1 to the NCBI nr database using Blast (e-value <1e-05), resulting in 7396 non-identical sequences, including sequences from HIV-1, HIV-2, and SIV. Identical sequences and fragments that cover less than 90% of whole-length Gag protein were removed. The remaining 7396 sequences were aligned using MUSCLE [23] using default parameters and ‘-maxiters 2’ to speed up the computations. The annotation of sequences to a certain virus strain was inherited from the NCBI annotation. The alignment is available in File S1.

### Identification of Co-evolving Positions

We computed co-evolving positions in a manner most similar to ref. [3], adding an evaluation of statistical significance. For each pair of positions *i* and *j* of the multiple sequence alignment, a correlation measure was calculated as:


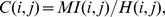


where 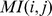 is the mutual information of the two positions, and 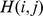 is their joint entropy, being calculated as:


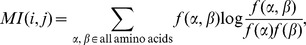



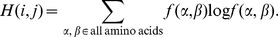


The amino acid frequencies were corrected to account for similarities of physico-chemical properties of the residues:


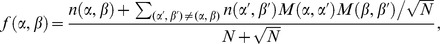



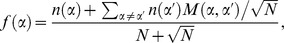


where 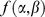 and 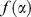 are the frequencies of amino acids *α* and *β* in positions *i* and *j* or of the amino acid *α* in position *i*, respectively, *n*(…) are the respective counts, and 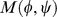 is the probability of substituting amino acid *φ* to amino acid *ψ* according to the BLOSUM62 matrix, *N* is the number of sequences in the alignment. Although there are specific matrices for substitution in HIV *pol*, *pro* and *env* [24], it is not known whether they apply to *gag*. The counts were corrected for differences in phylogenetic distance by multiplying by each by a weight term defined by the Gerstein-Sonnhammer-Chothia weighting scheme [25]. Gerstein-Sonnhammer-Chothia weights were calculated, as laid out in detail in [25], based on pairwise sequence identity, such that sequences that have higher identity receive a lower weight, and distant sequences receive high weights. So, a bunch of closely related sequences would make a contribution to the final count comparable to a contribution of a single distant sequence, accounting for over-representation of certain too close strains in our dataset.

All pairs of positions were ranked by descending correlation score 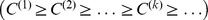, and a threshold k* in this list was set, following ref. [26], to select to the number of correlated pairs least probable to arise by chance:


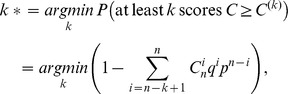


where *n* is the total number of pairs considered, 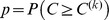 assuming the normal distribution of *C*, 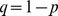.

### Infectivity Assay

Proviral plasmids were derived from pNL4–3 [27], and carried the single change 301F or the double change 203D, 301F inserted by PCR mutagenesis and confirmed by sequencing. Corresponding virus preparations were obtained by transfection of 293T cells [28] were analysed for infectivity on TZMbl cells [29] as described. Infectivity was scored as relative light units due to HIV Tat-dependent production of luciferase in TZMbl cells and was normalized for input virus. The graph shows mean and standard deviation of 2 independent experiments, each performed in triplicate. Transfection and titration of wild type HIV-1 and the two variants were performed in parallel.

## Supporting Information

File S1 **Alignment of Gag sequences used in this study.**
(GZ) Click here for additional data file.
